# Bibliometric Analyses Reveal Patterns of Collaboration between ASMS Members

**DOI:** 10.1007/s13361-017-1846-1

**Published:** 2018-01-05

**Authors:** Magnus Palmblad, Nees Jan van Eck

**Affiliations:** 1 Center for Proteomics and Metabolomics, Leiden University Medical Center, Leiden, The Netherlands; 2 Center for Science and Technology Studies, Faculty of Social and Behavioral Sciences, Leiden University, Leiden, The Netherlands

**Keywords:** ASMS

## Abstract

We have explored the collaborative network of the current American Society for Mass Spectrometry (ASMS) membership using bibliometric methods. The analysis shows that 4249 members are connected in a single, large, co-authorship graph, including the majority of the most published authors in the field of mass spectrometry. The map reveals topographical differences between university groups and national laboratories, and that the co-authors with the strongest links have long worked together at the same location. We have collected and summarized information on the geographical distribution of members, showing a high coverage of active researchers in North America and Western Europe. Looking at research fields, we could also identify a number of new or ‘hot’ topics among ASMS members. Interactive versions of the maps are available on-line at https://goo.gl/UBNFMQ (collaborative network) and https://goo.gl/WV25vm (research topics).

**Electronic supplementary material:**

The online version of this article (10.1007/s13361-017-1846-1) contains supplementary material, which is available to authorized users.

**Graphical Abstract:**
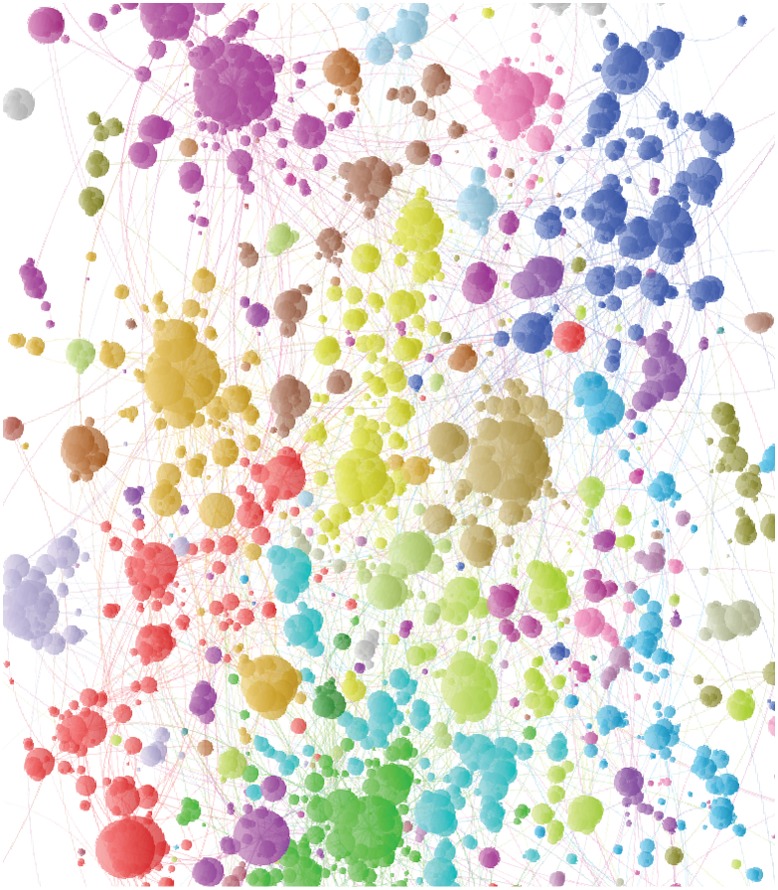
ᅟ

## Introduction

As a scientific society, the ASMS has an obvious interest in the collaboration among its members and how such collaboration and mentoring influence the development of the field of mass spectrometry. The history of mass spectrometry is addressed by many perspective articles and several books on the topic [1, 2]. The high degree of connectivity between past and current researchers in the field was recently made clear in two historical perspectives volumes of the Encyclopedia of Mass Spectrometry [3, 4]. While in no way a substitute for the historical research, contexts, and narratives provided in these volumes, systematic studies of the scientific literature can complement and illustrate past and present collaborative patterns. Such investigations are common in the field of bibliometrics [5], which can be defined as the study of bodies of interrelated documents, for example the scientific literature. Bibliometrics provides an established methodology for analyzing and visualizing connections among research developments and perhaps about the structure of the leading research groups and laboratories in the field.

Here we present the results from an analysis of the published literature looking specifically at a network of collaborations between members of the ASMS. A preliminary version of this network was presented as a poster at the 2016 Annual ASMS Conference in San Antonio, TX. In this paper we report additional layers of information on research topics and geographical locations of authors. From feedback received at the conference, we have also added additional quality control measures to capture as much of the past collaboration between ASMS members as possible, without including too many unrelated publications from ambiguous non-member namesakes. We also investigate relative positions in the author lists.

## Methods

### Membership Data

In this work, we define the ASMS membership as all 10,011 persons who were members at any time in the period from October 21, 2015 until October 14, 2016. Specifically, we received the member register from the ASMS and merged the unique member ID, last name, first name, e-mail address, full name, company, and journal address consisting of two lines followed by city, state, country, and postal code for all members on October 21, 2015, January 29, May 20, and October 14, 2016. All information was provided as supplied by the members. The ID, last name, first name, and full name were supplied for all 10,011 members. Company information was provided for 9040 members with at least a partial journal address for all members, although the city sometimes appeared in a field other than the designated city field. Since the information was entered by the members in free text fields rather than selected from a list, we also ‘cleaned up’ the affiliations by combining equivalent descriptions. In total, 26 regular expressions were used to correct the address fields and to provide member-specific missing information. The edited affiliation information was then used to disambiguate members from other authors of the same name.

### Identification of Publications of ASMS Members

In our analysis, we use data from the in-house version of the Clarivate Analytics Web of Science (WoS) database (http://wokinfo.com/) available at the Center for Science and Technology Studies at Leiden University. We take into account all WoS-indexed publications of the document types, article, letter, and review that appeared in the period 1980–2015 (the years available in our WoS database).

The ‘cleaned’ affiliation data mentioned above was used to match ASMS members with authors in the WoS database. Because of ambiguity in author names, this is a challenging task. One difficulty is the problem of synonyms, that is, the name of an ASMS member may appear in different ways in different publications. For example, John R. Yates appears in some publications as ‘Yates, JR’ and in others as ‘Yates, J’. There can be various reasons for the presence of synonyms, including name changes due to marriage, different standards adopted by scientific journals for presenting author names, variations in transliterations of names in non-Roman alphabets, and typographical errors. A second difficulty is the problem of homonyms, that is, the name of an ASMS member may not be unique. For example, the name ‘Smith, RD’ appears in more than 2000 publications in the WoS database, but only a subset of these publications have been written by Richard D. Smith affiliated with the Pacific Northwest National Laboratory in the United States. This problem is especially serious for Asian names.

To deal with the above problems, an algorithm for author-name disambiguation was used. This algorithm has identified authors in the WoS database at a high level of accuracy [6]. Using an iterative rule-based matching approach, we matched the ASMS members to the authors identified by our author-name disambiguation algorithm. Several matching steps were performed. Each step included only members who had not been matched in earlier steps. The matching approach started by applying the most restrictive matching rules (e.g., exact match on last name, first name, e-mail address, organization name, country, city, and scientific field) and then proceeded with less restrictive matching rules (e.g., match on last name, first initial, full name, country, and ‘fuzzy match’ on organization name). The less restrictive matching rules were used to obtain matches despite the presence of inaccuracies or inconsistencies in the data. For example, they allowed ASMS members to be matched to authors in the WoS database despite some data elements for the ASMS members being missing (e.g., the e-mail address) or despite inconsistencies between the ASMS member data and the WoS database (e.g., inconsistencies in first names or organizational names).

Using the approach described above, we managed to identify the WoS-indexed publications of 5650 ASMS members. There are 221,511 WoS-indexed publications that have been co-authored by one or more of these 5650 ASMS members. However, since our focus is on collaboration between ASMS members, our analysis takes into account only the 30,937 publications that have been co-authored by at least two ASMS members. We note that our focus on publications co-authored by multiple ASMS members also decreases the probability of our analysis being affected by errors in the matching of ASMS members with authors in the WoS database. If an ASMS member has been matched with an incorrect author in the WoS database, it is unlikely that this ASMS member will have co-authored with other ASMS members, and therefore the error in the matching will not lead to errors in the analysis.

### Member Co-Authorship Network

There are 1124 ASMS members who have not co-authored with any other ASMS member. These ASMS members have not contributed to the 30,937 publications on which our analysis is based, and they were therefore excluded from the analysis. The 30,937 publications resulted in a co-authorship network of 5650 – 1124 = 4526 ASMS members. This network turned out to consist of multiple connected components. Our analysis focuses exclusively on the largest connected component of the co-authorship network. The largest connected component includes 4249 ASMS members. In the rest of this paper, when we mention the co-authorship network, we refer to the largest connected component of this network.

There are 17,222 pairs of ASMS members who have co-authored at least one publication. Some of them of course have co-authored multiple publications, and therefore the total number of co-authorship links equals 60,476. The VOSviewer software tool [7, 8] was used to visualize the co-authorship network. VOSviewer, developed by van Eck, is a popular software tool for visualizing bibliometric networks. It is freely available at www.vosviewer.com. In the visualization of the co-authorship network, the size of the symbol representing an ASMS member was determined by the number of co-authored publications with other members. When visualizing a network using VOSviewer, suitable values need to be chosen for a number of technical parameters. In order to obtain a high-quality visualization of our co-authorship network, the attraction and repulsion parameters of the layout technique used by VOSviewer were set to 1 and –1, respectively. The resolution parameter of the clustering technique was set to its default value of 1. The locations of the members in the visualization were determined in such a way that individuals who have co-authored with each other tend to be located close to each other in the visualization. Co-authorship links were also used to group the members into clusters of individuals who are relatively strongly connected with each other. In the visualization of the co-authorship network, the color of a name indicates the cluster to which the member belongs.

### Term Co-Occurrence Network

We also created a visualization of a term co-occurrence network. To create this visualization, we started by analyzing the titles and abstracts of the above-mentioned 30,937 publications using natural language processing techniques [9]. For each publication, the noun phrases occurring in the title and abstract of the publication were identified. Of all 3597 noun phrases that were found in at least 30 publications, the 2500 noun phrases that appeared to be most relevant were algorithmically selected. We refer to these noun phrases as terms.

For each pair of terms, we counted the number of publications in which the terms occur both in the title and abstract. In this way, a term co-occurrence network was obtained. This network was also visualized using the VOSviewer, with the attraction and repulsion parameters of the layout technique set to the values of 1 and 0, respectively. The resolution parameter of the clustering technique was set to its default value of 1. In the visualization of the term co-occurrence network, the size of a term reflects the number of publications in which the term occurs, and the distance between two terms provides an approximate indication of the relatedness of the terms. The relatedness of terms was determined based on their number of co-occurrences. Hence, the larger the number of publications in which two terms both occur, the stronger the relation between the terms and the smaller, on average, the distance between the terms in the visualization. Colors represent clusters of terms that are relatively strongly related to each other.

### Geographical Distribution of ASMS Members

As it may be of interest to the society and its members, we also generated summary statistics on the geographic distribution of members based on the ‘cleaned’ journal address data. Member locations (city and country) were mapped to geographical coordinates and projected onto an OpenStreetMap world map using Tableau Desktop Public Edition ver. 10.0 (Tableau Software, Seattle, WA, USA).

## Results and Discussion

### General Observations

**Figure 1:**
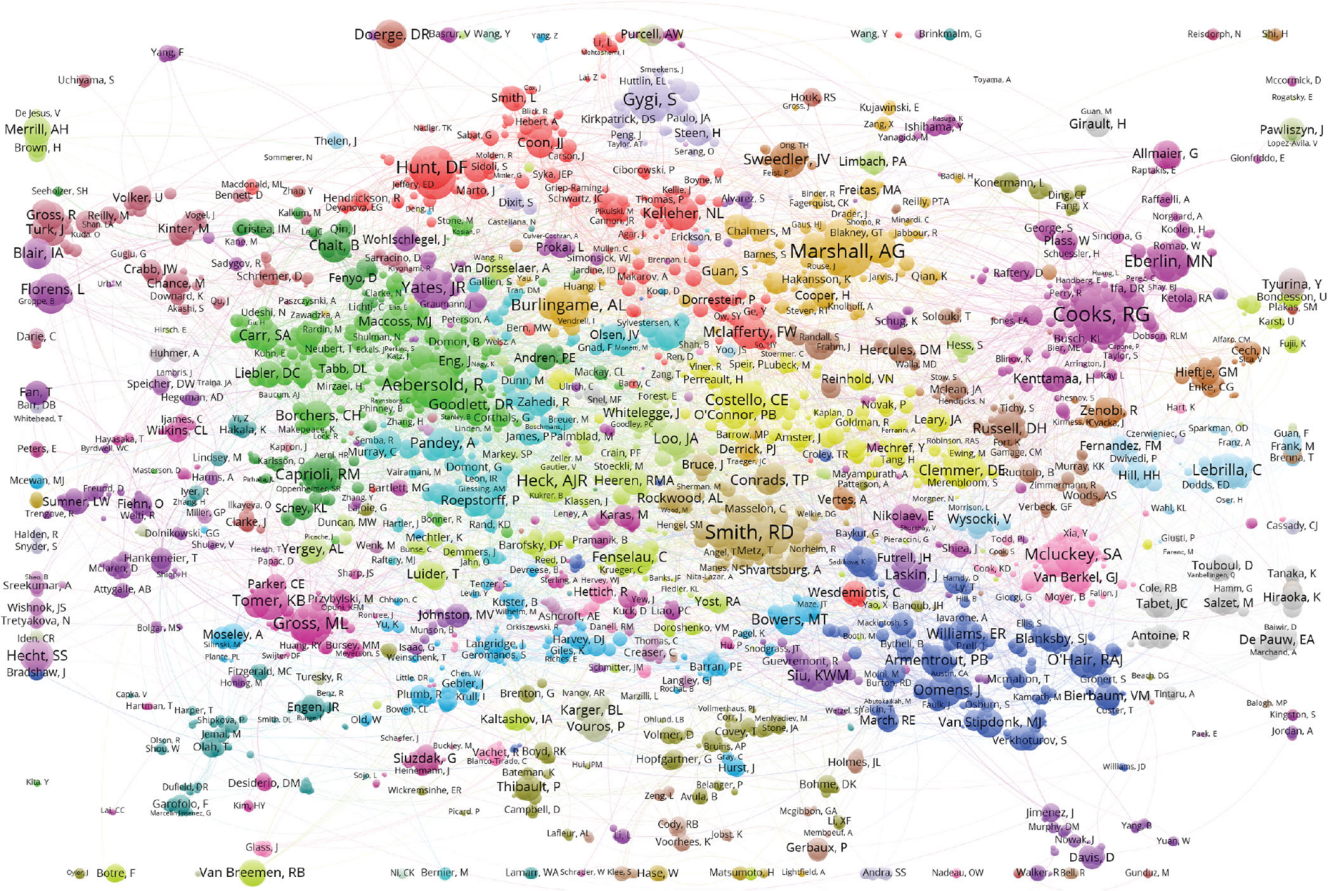
The ASMS membership collaborative network. This map is the primary result of the study. Names far from the center were projected to the rim of the map. The original and complete network can be interactively explored using the VOSviewer on https://goo.gl/UBNFMQ

**Figure 2:**
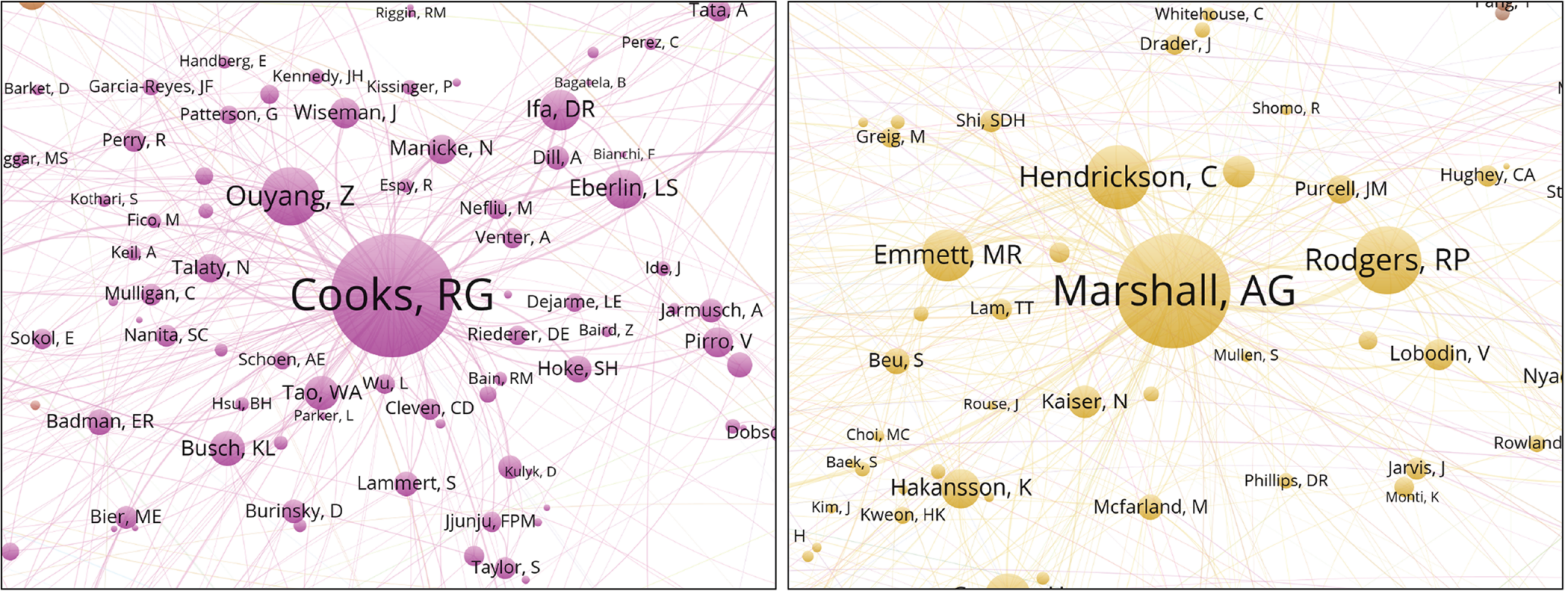
Examples of university (left) and national lab (right) coauthorship clusters. The single-university clusters are dominated by a central node (PI) surrounded by mostly small nodes corresponding to current and former graduate students and postdocs. Contrastingly, the national laboratory clusters tend to have a small number of medium-sized nodes corresponding to senior (permanent) staff surrounding one central author

**Figure 3:**
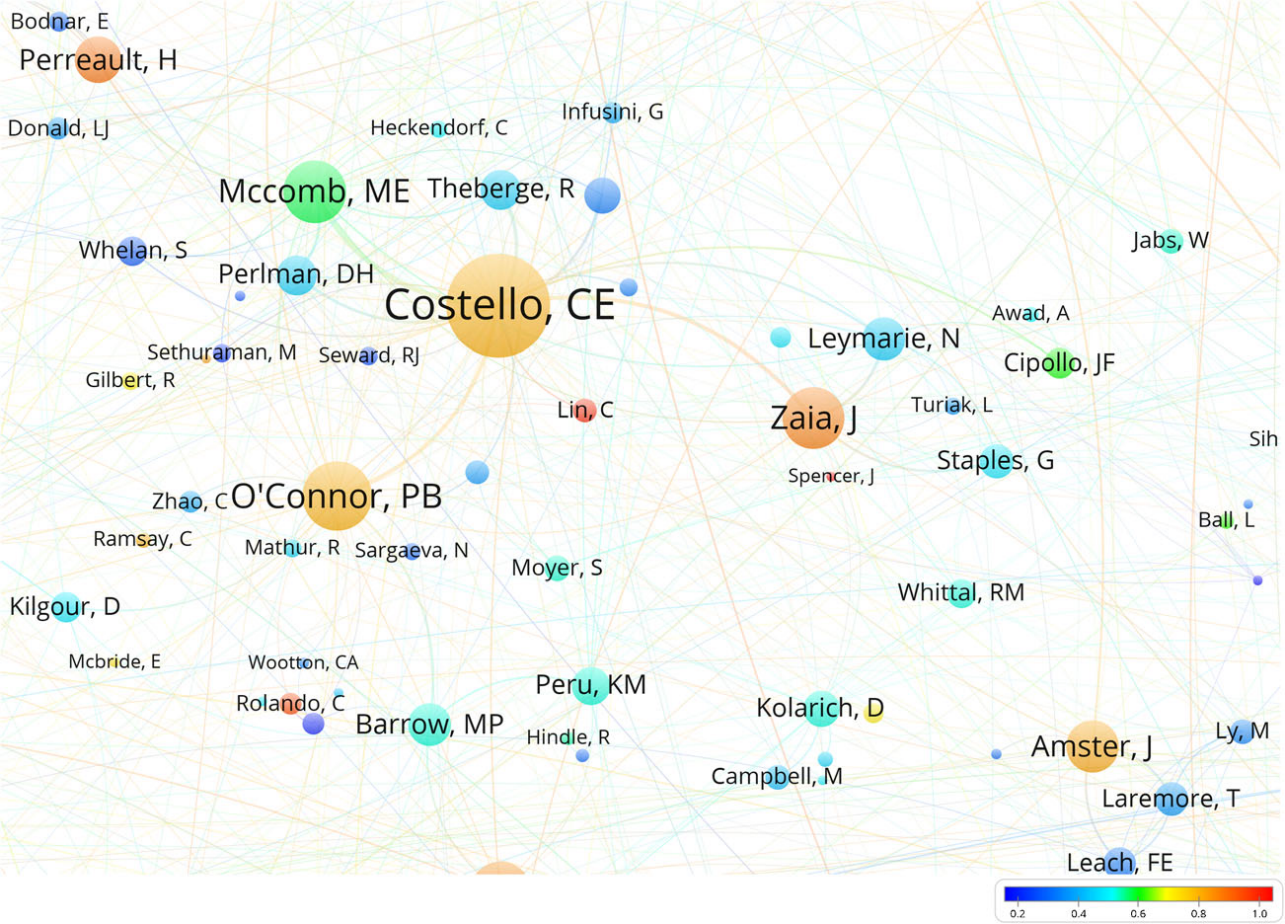
The normalized author order (scale bar) introduced in this paper helps distinguish infrequent but senior collaborators (red) from junior staff such as Ph.D. students or postdocs (blue) and provides another means to identify principal investigators in complex collaborative clusters. All ASMS members except Kerry M. Peru in this map region belong to the same cluster

In the co-authorship network (Figure 1), we can immediately observe a difference between single university co-authorship clusters, such as Purdue (Cooks) and large National Laboratory clusters, such as the National High Magnetic Field Laboratory (MagLab) (Figure 2). The university clusters are dominated by a central node (principal investigator or lab head) surrounded by many small nodes, corresponding to current and former Ph.D. students and postdocs. The national laboratory clusters instead appear to have a small number of medium-sized nodes corresponding to senior (permanent) staff, sometimes surrounding one central author (Alan G. Marshall at the MagLab, Richard D. Smith at Pacific Northwest National Laboratory). An overlay visualization is also available, showing for each author the average position in the author list of the publications of the author (Figure 3). In a deeper analysis, it may be possible to predict the job function or career stage of a member based on mean normalized authorship order, as Ph.D. students and junior postdocs typically appear early in author list, technical/support staff in the middle, and the PI or group leader often at the end.

Pairs of members having a known and long history of local collaboration unsurprisingly have the strongest link, e.g. D.F. Hunt and J. Shabanowitz, J.A. and R.R. (Ogorzalek) Loo, L. Florens and M. Washburn, and L.J.M. Dekker and T.M. Luider. The absence of geographically distant pairs with such a strong link suggests long-distance collaborative relationships are less easy to establish or maintain, at least in this field.

The requirement of having two (current) ASMS members as co-authors turned out to be a very strict filter on WoS publications – reducing the 274,197 papers on mass spectrometry with at least one member matching a name in the author list to 30,937 papers with at least two members found among the authors. This has the advantage of showing only true collaborative connections between recent members of the ASMS. It should be kept in mind that the size of the nodes in the collaborative map is proportional to the number of papers co-authored with other recent or current members of the ASMS, not to the total number of publications or publications with former members of the society. This most strongly affects senior researchers who are already clearly visible in the map. The six current ASMS members with the highest total number of publications are R.G. Cooks, J.R. Yates, R.D. Smith, R. Aebersold, S.S. Hecht, and A.G. Marshall. However, the *fraction* of publications co-authored with other ASMS members varies greatly, from 84% (Marshall), 73% (Smith), and 67% (Cooks) to 37% (Yates) and 26% (Hecht). The map is mass spectrometry centric, emphasizing research and development of mass spectrometry technology, which is often done in collaboration with other ASMS members, over adaptation or application of the technology in chemistry or biology together with scientists less likely to be members of the society.

Those familiar with the field may miss a few well-known names in the co-authorship map. The reason for this is that none of these is a current or recent member of the society. Absent from the map are also retired or deceased former members. This eliminates some recent ‘giants’ in the field who were long-term ASMS members. However, arbitrarily inserting some but not all former members would bias subsequent analyses. Information of all former members was not available to the authors. In some cases, these missing names may explain the more diffuse clusters that lack an obvious central node. Conversely, removing a central node from an existing cluster does not make the cluster disappear: it only becomes more diffuse.

### Coverage of the Scientific Literature

A majority of the recently most active researchers in the field of mass spectrometry are also members of the ASMS, including six of the 10 most published authors on the topic. Out of 109,223 papers in Web of Science on mass spectrometry (matching the search string “mass spectrom*” in the title, abstract, or author keyword fields) published from January 1, 2010 until December 31, 2015, 13,998 included at least one and 7731 at least two ASMS members. The ASMS members and their research output can therefore be assumed to cover most developments in the field and be representative for the field as a whole over the last three decades.

### Geographic Coverage

**Figure 4:**
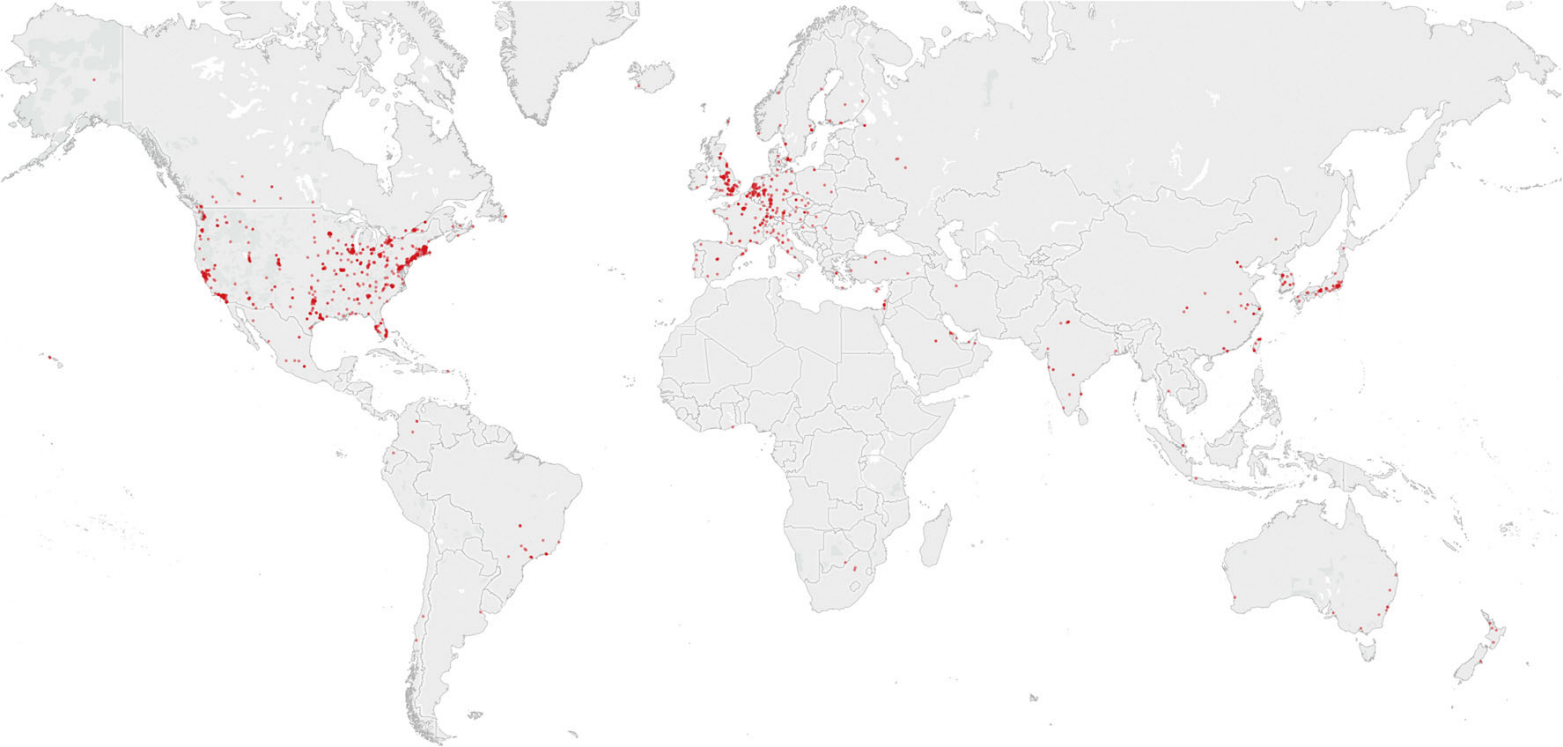
Geographical location of 8896 ASMS members resolved by the Tableau geoparser. Darker colors indicate multiple members with the same location (city and country). The underlying cartography is © OpenStreetMap contributors (http://openstreetmap.org) and open data licensed under the Open Data Commons Open Database License

The geographic distribution of ASMS also shows some interesting patterns (Figure 4). Based on the country information provided by the members themselves in their journal address, including members having opted out of receiving the print copy of JASMS, we find that 7095 or 71% of members of the ASMS are based in the United States (excluding Puerto Rico) and another 492 or 5% in Canada. This is perhaps not surprising giving the name and history of the society. However, a fairly large number of members, at least per capita, come from many Western European nations, including the UK (407 members), Germany (308), Switzerland (128), France (120), The Netherlands (97), Denmark (83), and Sweden (58). There are also many members from Asia-Pacific countries, such as Japan (209), China (194), Australia (106), South Korea (106), and Taiwan (87). Two countries with considerable research activity in the field of mass spectrometry but perhaps unexpectedly small number of ASMS members are Brazil (40 members) and Russia (only 20 members). This may be explained by a combination of factors, such as the travel required to attend the ASMS annual meeting and presence of active national mass spectrometry societies. It should be noted that ASMS is very different from the International Mass Spectrometry Foundation (IMSF), the successor to the International Mass Spectrometry Society [10]. The IMSF is formally registered as a non-profit foundation in The Netherlands and serving as an umbrella organization for 42 affiliate national mass spectrometry societies (with the ASMS representing the United States). The IMSF does not have a membership body of individual researchers like ASMS. It can therefore be argued that the lack of a comparable international society bestows on the ASMS an informal status of also being the *de facto* international society of researchers who are active in the field.

The ‘cleaned’ journal addresses (city and country) can be geoparsed to latitudes and longitudes by Tableau for 88.9% or 8896 of the 10,011 members. The resulting geographical distribution is shown in Figure 4. Hotspots can be observed in Boston and the San Francisco Bay Area, but also St. Louis, MO, Minneapolis, MN, and Raleigh, NC. Globally we can also observe a high density of ASMS members in the UK (especially around Manchester), southern England, Paris, and in an arc stretching from the Low Countries through western Germany to Switzerland and northern Italy. These are historical regions of industry and home to many research institutions and universities. In Asia, the Greater Tokyo and Osaka/Kyoto Areas have the highest concentration of ASMS members. It is not surprising many members are found at major universities and industries, but geospatial analysis also reveals regions with a high activity in the field of mass spectrometry relative to overall research output, such as Richland, WA, and St. Louis, MO.

### Research Topics

**Figure 5:**
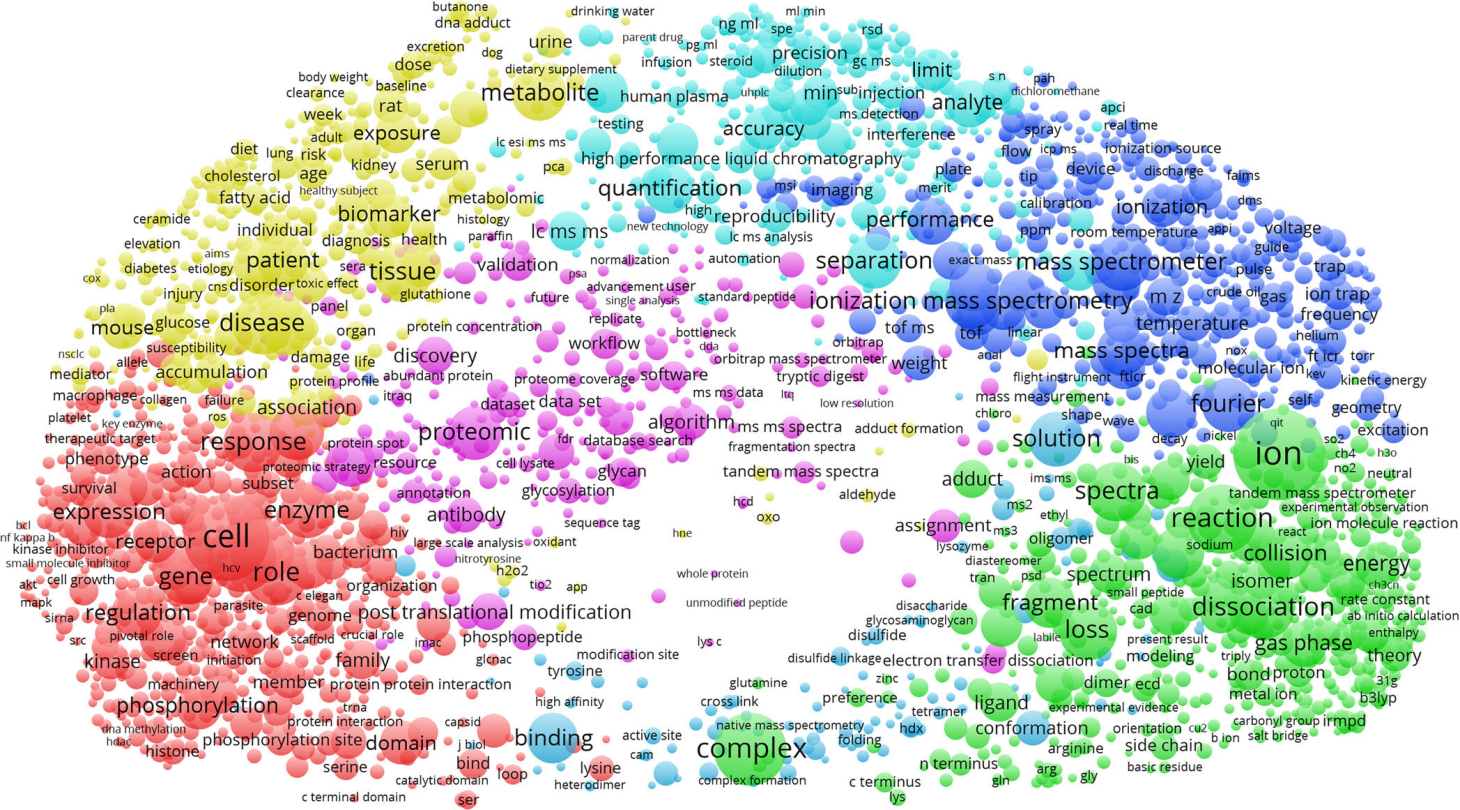
Research topics clustered by co-occurrence of terms in publications of ASMS members. The seven clusters can be broadly interpreted as clinical chemistry (cyan), mass spectrometry instrumentation (blue), gas-phase chemistry (green), structural biology (blue), cell biology (red), proteomics bioinformatics (magenta), and biomedicine (yellow). The term map can be further investigated by launching the VOSviewer from https://goo.gl/WV25vm (note the “Overlay Visualization” tab)

**Figure 6:**
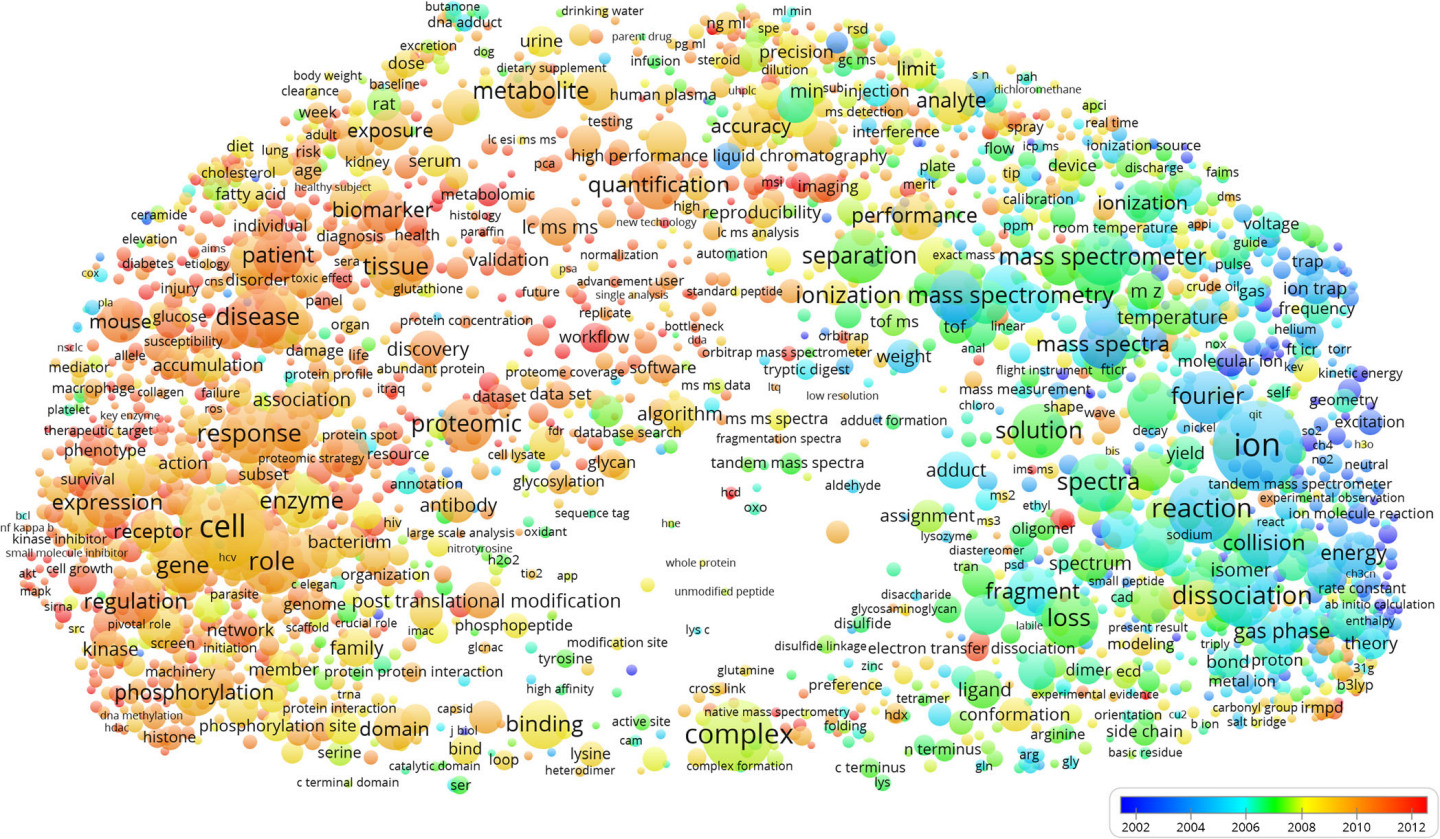
Research topics clustered as in Figure 5, but overlaid with average publication year (scale bar). We used average publication year as a scalar proxy indicating how ‘new’ or ‘old’ (or more positively, ‘mature’) a particular research topic is among ASMS members

The ASMS members co-publish on a wide range of research topics, from fundamental ion and gas-phase chemistry, mass spectrometry instrumentation and applications in clinical chemistry, biomedical research, cell biology, and proteomics/bioinformatics (Figure 5). By overlaying the average publication year (Figure 6), we see that biomedical applications, clinical chemistry, cell biology, and proteomics are relatively newer or ‘hot’ (average publication year around 2010), and fundamental chemistry and instrumentation relatively older or ‘cold’ (average publication year around 2002). More interesting is perhaps to look for local cold- and hotspots within the topical clusters. We then notice that “cDNA”, “Edman degradation” and all terms related to 2D-PAGE are relatively old in the cell biology and proteomics clusters. Conversely, electron transfer dissociation and ion mobility are hot topics in gas-phase/ion chemistry and instrumentation, respectively (electron transfer dissociation is assigned to the proteomics cluster, but it is localized within the gas phase/ion chemistry cluster by the VOSviewer). The overlay visualizations also include geographical (country) bias in research topics for the most frequently occurring countries. We note a positive bias toward the mass spectrometry instrumentation cluster for Germany and Japan, two countries also home to major manufacturers of mass spectrometry equipment.

## Conclusions

To our knowledge, this is the first comprehensive bibliometric and co-authorship analysis of an entire international scientific society. A number of non-trivial challenges had to be overcome in the matching of member names and addresses with unique authors and affiliations in Web of Science. As members or their co-authors retire, the collaborative map changes. It is tempting to speculate that such former members can explain some of the diffuse co-authorship clusters seemingly lacking a central node.

The collaborative network of ASMS members shows clear geographical patterns of collaborations. In particular, the co-authors with the strongest link have all shared the same affiliation for an extended period of time, often decades. As largely an experimental field using often large and expensive instrumentation, it is also possible that this geographical component is stronger than in other research domains.

Co-authorship analysis within a single field – mass spectrometry – captures some of the differences between the principal investigator-centered research in academia with the team-based ‘big science’ at the national laboratories. A majority of the most published researchers in the field of mass spectrometry are also members of the ASMS. This is particularly true for North America, but also for Western Europe.

## Electronic Supplementary Material

ESM 1 (PNG 7.74 mb)
