# Supplementary figures and images for: Bibliometric Analyses Reveal Patterns of Collaboration between ASMS Members
Source: J Am Soc Mass Spectrom. 2018 Jan 5;29(3):447–54. doi: 10.1007/s13361-017-1846-1 (PMC5838206; doi:10.1007/s13361-017-1846-1)

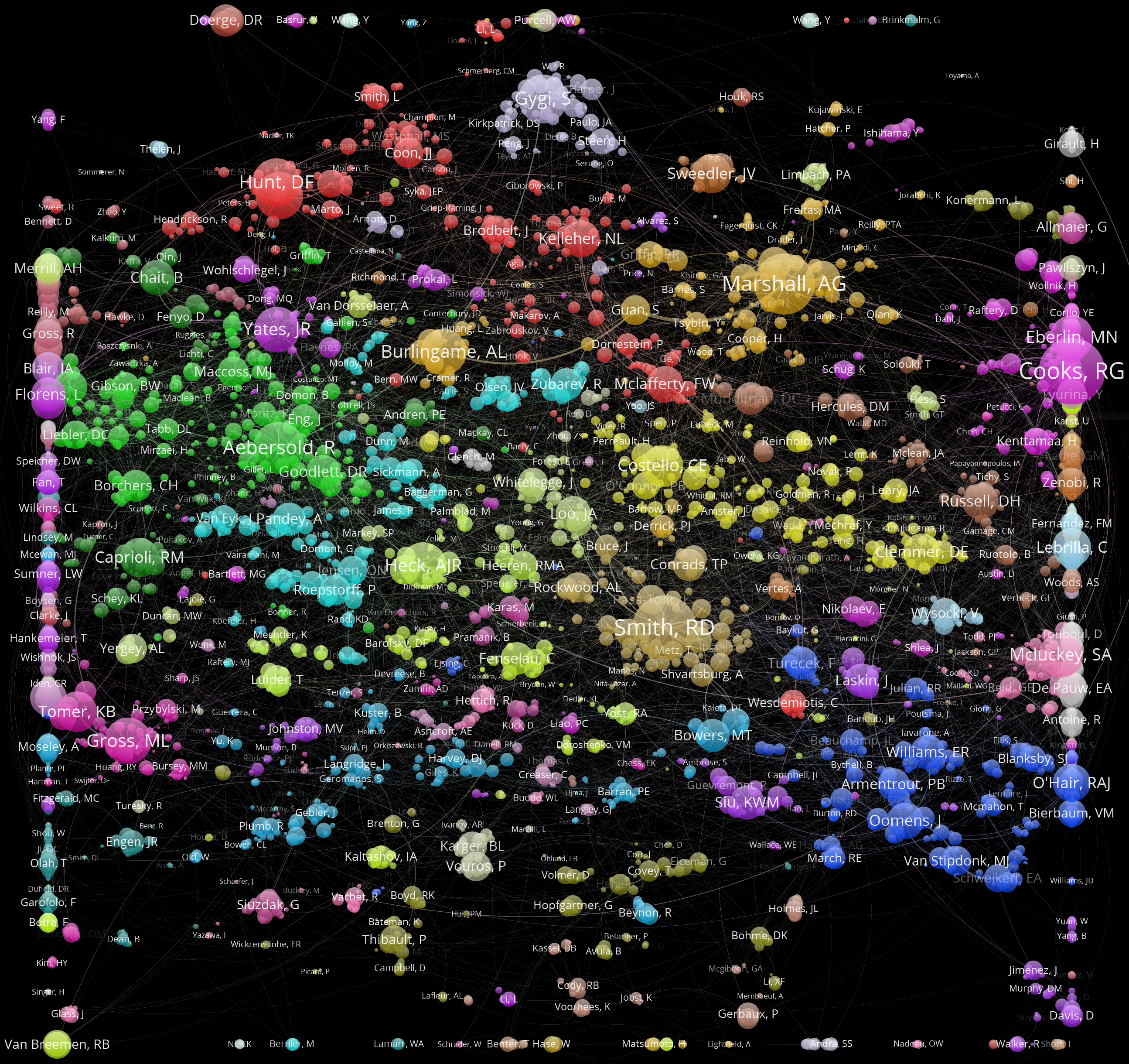

Supplement: Supplementary file 1 — (PNG 7.74 mb) [file 13361_2017_1846_MOESM1_ESM.png]
